# Tropical Data: celebrating 10 years of impact on the global trachoma programme

**Published:** 2026-08-31

**Authors:** Emma Harding-Esch, Cristina Jimenez, Ana Bakhtiari

**Affiliations:** 1 Chief Scientist: Tropical Data, London School of Hygiene & Tropical Medicine, UK.; 2 Programme Manager: Tropical Data, Sightsavers, Haywards Heath, UK.; 3 Director, Data and Analytics: Tropical Data, International Trachoma Initiative, Decatur, Georgia, USA.

**Standardised, country-owned data support health ministries to achieve trachoma elimination.**

**Figure:**
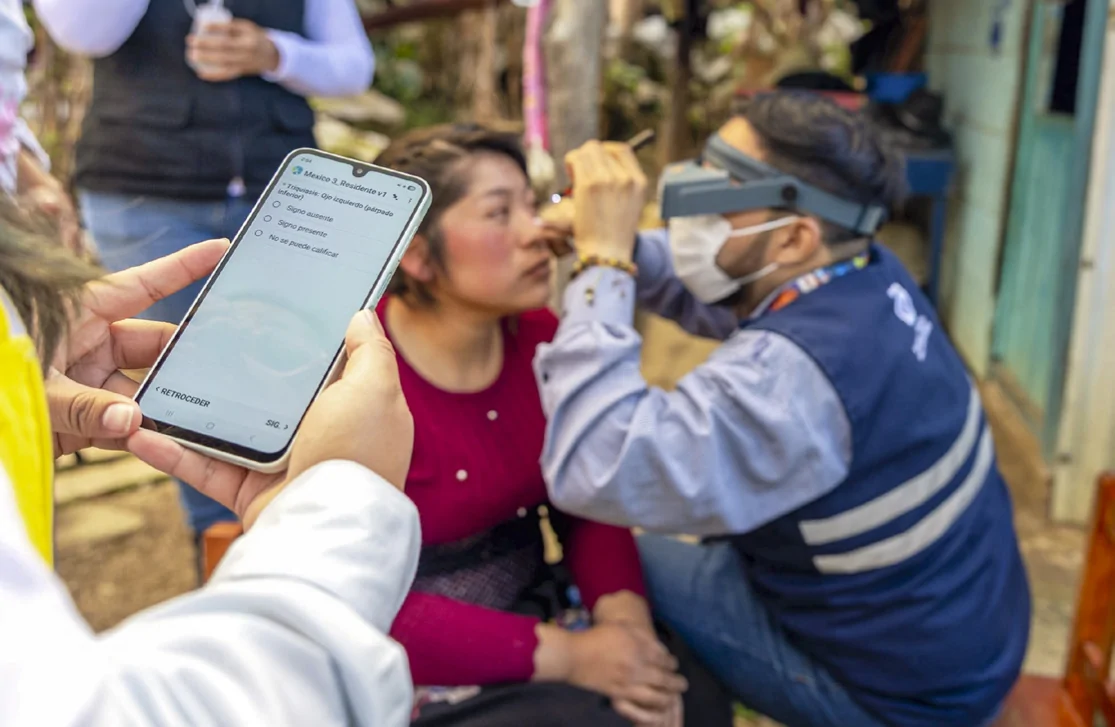
A person being examined during post-validation surveys. MEXICO

© A IVÁN LÓPEZ, OPS/OMS CC BY-NC-SA 4.0

Significant progress is being made to eliminate trachoma as a public health problem. The number of people at risk fell from 1.5 billion globally in 2002 to 97.1 million in November 2025 – a 94% reduction. As of August 2026, 32 countries have been validated by WHO as having eliminated trachoma. These achievements are the result of sustained collaboration between health ministries, international partners, donors, researchers, and communities, underpinned by high-quality data.

Methodologically robust, standardised data have been central to this progress. Knowing where trachoma is endemic, where interventions are required, and when elimination thresholds have been reached are essential for informing national programmes and maintaining momentum toward elimination. WHO launched Tropical Data in 2016, building on the methods and technologies developed as part of the Global Trachoma Mapping Project (2012–2016), at that time the largest infectious disease mapping effort ever undertaken. Tropical Data initially consisted of a consortium of partners, including the International Trachoma Initiative (ITI), London School of Hygiene & Tropical Medicine, RTI International and Sightsavers, with core funding currently provided by ITI and Sightsavers.

Tropical Data (www.tropicaldata.org) is a free service that provides health ministries with comprehensive support throughout the entire survey process, from protocol development and training, to data cleaning, analysis, and the approval of results. Tropical Data team members help countries to follow WHO-recommended methodologies, which produces epidemiologically robust data that can support programmatic decision-making and can be included in dossiers submitted to WHO to support trachoma elimination claims. This standardisation makes it possible to track progress over time and to compare results both within and between countries, so that interventions can be delivered where they are needed most.

At the core of Tropical Data's work is an emphasis on country ownership. Health ministries lead the survey process and retain full ownership of their data, while receiving technical support from Tropical Data. This approach strengthens national capacity in a range of areas, including protocol development and training; it also builds sustainable expertise and ensures that data translate into programmatic decisions. District-level prevalence categories are uploaded to the Trachoma Atlas (www.trachomaatlas.org) and ESPEN Portal for countries in WHO's African region (espen.afro.who.int). As well as showing the current distribution of trachoma, these open-access platforms can be used as advocacy tools and as a source of data for use in operational research. Health ministries can also choose to automatically share their point prevalence data with the joint ITI/WHO GET2020 database to help populate drug donation applications and trachoma dossiers.

Diseases like trachoma disproportionately affect people living in remote, marginalised settings where health information is often scarce. Initiatives such as Tropical Data help ensure that these communities are not overlooked, so that no one is left behind. For example, Tropical Data has worked with countries to develop methods that are feasible in settings affected by insecurity and conflict, some of which are highlighted in the ICTC publication ‘Delivering trachoma interventions to special populations: A toolkit for program managers’ (bit.ly/45WfTyQ). In addition, in order to support more cost-effective and comprehensive eye care approaches, Tropical Data has conducted research demonstrating the feasibility of integrating the collection of eye health indicator data into trachoma surveys.

Since 2016, Tropical Data has supported over 4,200 surveys across 55 countries, examining more than 13.7 million people. This represents one person examined for trachoma every 26 seconds. The survey results have helped guide the distribution of hundreds of millions of antibiotic treatments donated by Pfizer Inc., through ITI, leveraging billions of dollars of resources for the global trachoma programme. Equally important, survey results identify districts where interventions can be stopped, and highlight areas where further surveillance is needed. In many cases, data have confirmed that mass treatment is no longer required, enabling programmes to redirect resources elsewhere.

Tropical Data's success highlights what is possible when countries are equipped with the tools to generate, own, and use their data. It is a powerful reminder that in global health, evidence drives impact, and data can help turn the goal of eliminating trachoma into reality.

*For free support with trachoma surveys in your country, contact the Tropical Data team at* admin@tropicaldata.org

*To learn more about the history of Tropical Data, please visit* bit.ly/trop-hist

